# Capturing Change in Balance Confidence over 30 Days: Insights Gained from a Micro-Longitudinal Study

**DOI:** 10.1093/geroni/igab046.2895

**Published:** 2021-12-17

**Authors:** Tai-Te Su, Aileen Griffin, Faith-Christina Washington, Jacob Sosnoff, Shannon Meija

**Affiliations:** 1 University of Illinois at Urbana-Champaign, United States; 2 University of Illinois at Urbana-Champaign, Aurora, Illinois, United States; 3 University of Illinois at Urbana-Champaign, Champaign, Illinois, United States; 4 School of Health Professions, University of Kansas Medical Center, Kansas City, Kansas, United States; 5 University of Illinois, Champaign, Illinois, United States

## Abstract

Balance confidence reflects one’s estimate of their ability to maintain balance and avoid falls. Extensive literature has shown the relationships between balance confidence, functional limitations, and falls in later life. However, change in balance confidence, especially within short timescale, remains largely unknown and deserves further research. In this study, we aimed to investigate how older adults’ balance confidence would change over 30 days and explore whether baseline characteristics would explain the individual differences in change. We used data from the Daily Balance Project that employed intensive-repeated measurements to examine the dynamics of subjective and objective fall risk across a month. Twenty-one participants (age=78.6±5.8, 48%female) were enrolled, and individual characteristics were measured upon recruitment. Throughout the study, participants self-reported their daily balance confidence using the Activity-Specific Balance Confidence (ABC) Scale. We performed growth modeling techniques to examine change within a multilevel framework. Our results showed that overall, ABC scores were high (79.9±17.4) at first, but the linear change was non-significant (b=0.03, SE=0.21, p=.89) on average. However, we found that balance confidence increased in individuals with higher educational attainment (b=0.37, SE=0.13, p<.01) and decreased among those with greater physical fall risk (b=-0.18, SE=0.07, p<.01) and accurate understanding of fall risk at baseline (b=-0.24, SE= 0.12, p=.04). Although ABC scores were stable within the period of one month, our study highlights the distinction of individual characteristics in the process of balance confidence appraisal. We suggest that these nuances should be taken into account when developing more fine-grained fall risk assessments and interventions.

